# The effects of dietary net energy on grow-finish performance and carcass characteristics of market gilts managed with immunological suppression of ovarian function and estrus (Improvest)

**DOI:** 10.1093/tas/txae026

**Published:** 2024-03-01

**Authors:** Benjamin M Bohrer, Yifei Wang, Jose L Landero, Malachy Young, Blaine Hansen, D Steve Pollmann, Marnie A Mellencamp, Leanne Van De Weyer, Alvaro Aldaz

**Affiliations:** Department of Animal Sciences, The Ohio State University, Columbus, OH 43210, USA; Department of Animal Sciences, The Ohio State University, Columbus, OH 43210, USA; Gowan’s Feed Consulting, Wainwright, AB T9W 1L2, Canada; Gowan’s Feed Consulting, Wainwright, AB T9W 1L2, Canada; BCH Consulting LLC, Atlantic, IA 50022, USA; DSP Consulting LLC, Alpine, UT 84004, USA; Zoetis Inc., Parsippany, NJ 07054, USA; Zoetis Canada Inc., Kirkland, QC H9H 4M7, Canada; Zoetis Inc., Parsippany, NJ 07054, USA

**Keywords:** anti-GnRF, dietary energy, estrus suppression, immunocastration, pig production

## Abstract

The objective was to determine the effects of net energy (NE) during the grow-finish period on live performance and carcass characteristics of market gilts managed with immunological suppression of ovarian function and estrus (Improvest^®^; IMP) compared with market gilts not managed with Improvest (CON). The 104-d study began when 1,008 gilts (11 wk old; average starting weight of 30.8 kg) were allocated by weight to 48 pens with 21 gilts/pen. Half of the pens were randomly selected to be managed with Improvest while the other half of the pens were not managed with Improvest. Three dietary programs differing in their NE were formulated over five dietary phases (according to standardized ileal digestible lysine requirements) to provide an average of 2,218 kcal/kg (low NE), 2,343 kcal/kg (medium NE), or 2,468 kcal/kg (high NE). The experiment was designed as a 2 × 3 factorial with main effects of Improvest management and NE. For the overall study period, there were no significant interactions (*P* ≥ 0.20) for average daily feed intake (ADFI), average daily gain (ADG), or Gain:Feed (G:F). There were also no significant interactions between Improvest management and NE (*P* ≥ 0.30) for carcass characteristics. However, IMP gilts consumed more feed (6.8% greater ADFI; *P* < 0.01), grew faster (5.0% greater ADG; *P* < 0.01), were less efficient (1.8% lower G:F; *P* < 0.01), heavier (3.5 kg hot carcass weight; *P* < 0.01), and fatter (1.9 mm greater backfat thickness and 1.26% less predicted lean carcass yield; *P* < 0.01). No difference (*P* = 0.21) in carcass dressing percentage between IMP and CON gilts was reported. For the overall study period, gilts fed low NE and medium NE diets consumed more feed compared with gilts fed high NE diets (6.8% more ADFI for low NE and 5.7% more for medium NE; *P* < 0.01), and gilts fed low NE diets grew 2.5% slower (*P* < 0.01) than gilts fed medium NE diets, while gilts fed high NE diets were intermediate and not different from the other NE treatments. This resulted in gilts fed Low NE diets being the least efficient (3.8% lower G:F than medium NE and 7.1% lower G:F than High NE; *P* < 0.01). Overall, these data indicate that typical Improvest response levels were sustained at each of the NE treatments evaluated in this study as there were no significant interactions for Improvest management and NE; however, consideration should still be provided to the known production impacts of low NE diets.

## Teaser

This study evaluated the effect of dietary net energy (NE) in grow-finish pig diets when female pigs were managed with immunological suppression of ovarian function and estrus (Improvest). For the grow-finish period, pigs managed with Improvest consumed 6.8% more feed, grew 5.0% faster, and had 3.5 kg heavier carcasses. There were a limited number of statistically significant interactions in this study, indicating that typical response levels for important performance parameters can be sustained when feeding low NE diets to market gilts managed with Improvest.

## Introduction

Improvest^®^ (marketed under the trade name of Improvac^®^, Innosure^®^, or Vivax^®^ in some parts of the world; Zoetis Inc., Parsippany, NJ, USA) is a gonadotropin-releasing factor (GnRF) analog-diphtheria toxoid conjugate formulated product approved for the temporary suppression of ovarian function and the suppression of estrus in intact female pigs intended for slaughter. Managing market gilts with Improvest has several secondary production advantages when compared with conventional market gilts as a result of suppressing ovarian function, including 3% to 6% improvement in weight gain which results in 3 to 5 kg heavier hot carcass weights (HCW) and improved group uniformity (i.e., less light-weight pigs) among mixed-sex populations of pigs (Bohrer et al., 2014; Poulsen Nautrup et al., 2020; Vasquez-Hidalgo et al., 2023). Nutritional recommendations for market gilts managed with Improvest mirror that of physically castrated males and conventional market gilts; however, market gilts managed with Improvest consume 6.1% (95% confidence interval of 4.59% to 7.53%) more feed during the grow-finish period and 12.8% (95% confidence interval of 11.41% to 14.13%) more feed after the second dose of Improvest when compared with conventional market gilts (Vasquez-Hidalgo et al., 2022).

Energy is an important nutrient for growing animals and is the single most expensive component of swine diets on a total level of inclusion/consumption basis (Velayudhan et al., 2015; Noblet et al., 2022). Net energy (NE) of livestock diets is defined as the metabolizable energy of a diet after accounting for the heat of digestion, nutrient metabolism, and excretion of waste (Just, 1982; Zuidhof, 2019). The main source of energy found in North American swine diets is corn and/or corn byproducts; however, some regions of the world where corn is not commonly grown, such as western Canada, eastern Europe, and southern Europe, may utilize other grains such as barley or wheat for the main source of energy in swine diets (Woyengo et al., 2014; Velayudhan et al., 2015). Energy content of corn (NE = 2,672 kcal/kg) is approximately 15% greater than that of barley (NE = 2,327 kcal/kg) and is approximately 8% greater than that of wheat (NE = 2,472 kcal/kg; NRC, 2012). The NRC (2012) does not list dietary requirements for NE. The assumptions for NE content of the diet in grow-finish pigs, which is presented as a reference in dietary requirement tables, was 2,475 kcal/kg. In field studies conducted by PIC, high-energy grow-finish diets comprised of corn and high concentrations of oils have been reported to have NE levels as high as 2,733 kcal/kg and low-energy grow-finish diets comprised of barley and low concentrations of oils have been reported to have NE levels as low as 2,116 kcal/kg (PIC, 2021). It is understandable that crops grown in the given region of the world where pigs are raised will dictate the ingredients available with which a nutritionist may formulate diets; however, the interaction of these formulation constraints with emerging technologies is often not fully elucidated.

To this point, investigation of the impacts of NE level on live performance and carcass characteristics of market pigs is limited, even though several areas of the world feed pigs with ingredients with differing levels of NE. In particular, no current study exists comparing the effects of NE level for gilts managed with immunological suppression of suppression of ovarian function and the suppression of estrus. Therefore, the purpose of this study was to determine the effects of NE level during the grow-finish period on live performance and carcass characteristics of market gilts managed with immunological suppression of ovarian function and estrus (Improvest) compared with conventional market gilts.

## Materials and Methods

The experiment was conducted at a commercial research grow-finish facility located in Alberta, Canada. Pigs were managed in accordance with the Canadian Council on Animal Care (2009).

### Animals

At approximately 11 wk of age, 1,008 gilts (progeny from Duroc sires [PIC 800] × white line sows [PIC Camborough F1]; Genus PIC; Hendersonville, TN, USA) were weighed and allotted to a total of eight replications, each replication consisting of six different treatments (two Improvest management strategies and three dietary treatments). Each dietary treatment within replication was randomly assigned to one of 48 pens (6.1 × 2.4 m; 21 gilts/pen; initial stocking density of 0.7 m^2^/pig) in one room of the commercial grow-finish barn. Pens were equipped with a double-sided wet/dry stainless-steel feeder (Crystal Spring Hog Equipment; Agathe, Manitoba, Canada) and a single bowl drinker. The room was ventilated with negative pressure and was maintained within thermo-neutral temperatures for grow-finish pigs. Pigs had *ad libitum* access to feed and water throughout the duration of the grow-finish period.

### Administration of Improvest

One-half of the pens of gilts (*n* = 24 pens, eight replications, each replication consisting of three different dietary treatments) were administered Improvest [IMP; a GnRF analog-diphtheria toxoid conjugate product; Zoetis, Inc.] *via* injection by trained personnel according to manufacturer directions while the other half of the pens of gilts (*n* = 24 pens, eight replications, each replication consisting of three different dietary treatments) were not administered Improvest (CON). The first Improvest dose was administered on day 20 of the study and the second Improvest dose (which triggers the temporary suppression of ovarian function and estrus) was administered on day 55 of the study.

### Diets

**Figure 1.**
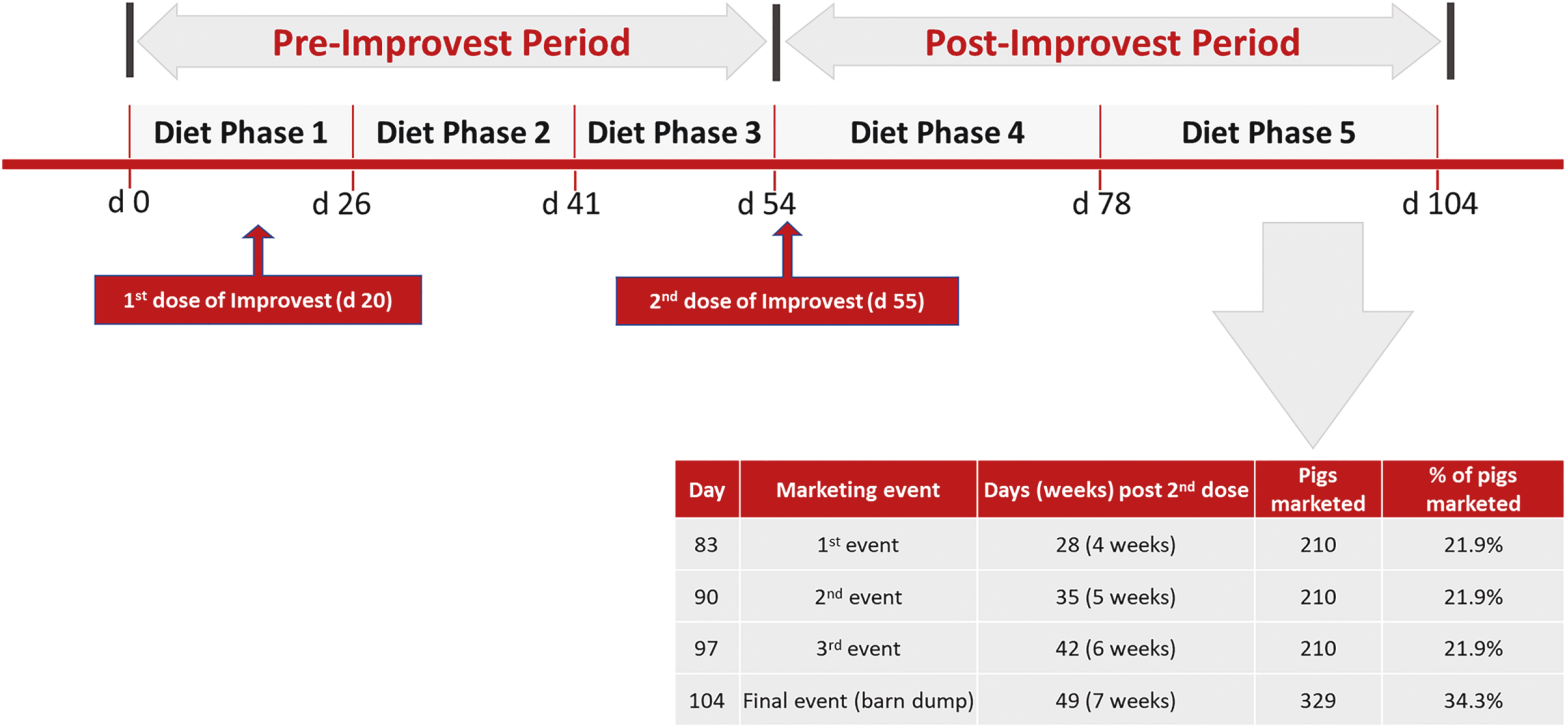
Timeline of important study events.

The experiment was conducted using five dietary phases: phase 1 (days 0 to 26 of the study; targeted weights of 31 to 58 kg); phase 2 (days 27 to 41 of the study; targeted weights of 58 to 75 kg); phase 3 (days 42 to 54 of the study; targeted weights of 75 to 89 kg); phase 4 (days 55 to 78 of the study; targeted weights of 89 to 117 kg); phase 5 (day 79 of the study to marketing; targeted weights of 117 to 135 kg; Figure 1). The second dose of Improvest was administered on day 55 of the study; therefore, dietary phases 1 to 3 aligned with the pre-Improvest period, and dietary phases 4 to 5 aligned with the post-Improvest period.

All diets were fed in the mash form and formulated with nutrient loading values for ingredients obtained from EvaPig® software (version 2.0.3.2; French National Institute for Agriculture, Food and Environment Research [INRAE], METEX Animal Nutrition, and French Association of Zootechnie [AFZ]) and verified using laboratory analysis. In brief, moisture was determined with AOAC methods 935.29 and 945.15, crude protein was determined with AOAC method 990.03, crude fat was determined *via* ether extraction with AOAC method Ba 3-38, starch was determined with AOAC method 996.11, crude fiber was determined with AOAC method Ba 6a-05/ Ba 6-84, acid detergent fiber was determined with AOAC method 973.18, neutral detergent fiber was determined with AOAC method 2002.04, and ash was determined with AOAC method 942.05 (AOAC, 2016). Digestible energy was calculated using the equation 1 to 3 from the NRC (2012): $\begin{array}{ll} & DE=4,168-(9.1\times ash)+(1.9\times crude\;protein)+(3.9\times \end{array}$$\begin{array}{ll} & ether\;extract)-(3.6\times neutraldetergent\;f\;iber) \end{array}$. NE was calculated using the equation 1 to 8 from the NRC (2012): NE=(0.700×digestible energy)+(1.61×etherextract) +(0.48×starch)−(0.91×crude protein)−(0.87×aciddetergent fiber).

In total, three dietary programs were fed in this study. Two of the dietary programs (low NE and high NE) were formulated and mixed at the mill, each of which met or exceeded nutrient requirements according to NRC (2012) for market gilts (Table 1). In addition to the low NE and the high NE diets, a 50:50 blend of the low NE and high NE diets (medium NE) was created using a robotic feeding system (FeedLogic; Wilmar, MN, USA) at the farm. The accuracy of the robotic feeding system was tested for each diet phase. This consisted of testing the accuracy with an on-farm standard operating procedure which consisted of manually weighing 15 kg samples of feed on a calibrated scale and ensuring accuracy was within 50 g (>99.5% accuracy).

**Table 1.** Composition of the low and high net energy (NE) diets formulated for gilts during each dietary phase^1^

	Phase 1 (days 0 to 26)		Phase 2 (days 27 to 41)		Phase 3 (days 42 to 54)		Phase 4 (days 55 to 78)		Phase 5 (day 79—marketing)	
	Low NE	High NE	Low NE	High NE	Low NE	High NE	Low NE	High NE	Low NE	High NE
Ingredient, %										
Corn	—	28.59	—	34.31	—	36.14	—	49.17	—	57.00
Barley	39.68	—	40.49	—	40.96	—	51.15	—	61.19	—
Faba beans	25.00	25.00	25.00	25.00	25.00	25.00	20.00	20.00	15.00	15.00
Wheat DDGS	15.00	15.00	15.00	15.00	15.00	15.00	10.00	10.00	5.00	5.00
Wheat	15.00	15.00	15.00	15.00	15.00	15.00	15.00	15.00	15.00	15.00
Soybean meal	2.33	10.43	2.00	5.36	2.00	4.20	2.00	2.09	2.00	4.88
Canola oil	—	3.00	—	2.57	—	2.40	—	1.54	—	1.10
Limestone	1.23	1.21	1.17	1.16	1.10	1.12	0.99	1.03	0.93	0.96
Salt	0.48	0.48	0.49	0.48	0.49	0.48	0.52	0.51	0.55	0.53
Monocalcium phosphate	0.30	0.32	0.13	0.21	—	—	—	—	—	—
Vitamin and mineral premix^2^	0.16	0.16	0.16	0.16	0.16	0.16	0.10	0.10	0.08	0.08
l-Lysine	0.492	0.464	0.362	0.450	0.218	0.322	0.188	0.350	0.196	0.286
l-Threonine	0.157	0.151	0.105	0.152	0.043	0.098	0.037	0.116	0.044	0.088
DL-Methionine	0.135	0.160	0.073	0.128	0.038	0.071	0.025	0.073	0.022	0.049
l-Tryptophan	0.037	0.041	0.017	0.042	—	0.023	—	0.032	—	0.024
Analyzed composition, dry matter basis (unless noted)										
Dry matter, %	89.90	92.38	89.20	92.05	91.22	92.33	91.31	91.09	90.47	90.26
Crude protein, %	24.5	23.1	24.4	21.8	22.4	20.9	19.7	18.2	18.3	14.7
Ether extract, %	2.02	4.47	2.30	4.23	2.23	4.07	2.52	3.71	2.53	3.49
Starch, %	41.0	40.7	41.9	42.6	47.6	46.5	48.7	53.7	50.5	58.7
Crude fiber, %	5.54	4.10	4.87	4.18	4.63	4.26	5.69	3.81	4.97	3.33
Acid detergent fiber, %	9.32	7.65	7.85	6.70	7.49	6.42	7.04	6.26	6.64	5.01
Neutral detergent fiber, %	15.8	12.7	14.7	11.2	13.8	12.9	14.8	10.6	13.8	10.2
Ash, %	4.62	4.17	4.80	4.46	4.11	3.60	4.10	3.75	4.05	3.18
Digestible energy (DE)^3^, kcal/kg as-fed	3,347	3,644	3,350	3,625	3,475	3,630	3,410	3,585	3,392	3,544
Net energy (NE)^4^, kcal/kg as-fed	2,276	2,542	2,298	2,552	2,428	2,581	2,418	2,598	2,427	2,626
Formulated values										
Net energy (NE)^4^, kcal/kg as-fed	2,200	2,450	2,200	2,450	2,200	2,450	2,230	2,480	2,250	2,500
Standardized ileal digestible (SID) lysine, % as-fed	0.98	1.09	0.87	0.97	0.76	0.84	0.68	0.75	0.63	0.70
SID lysine:NE, g/Mcal as-fed	4.44	4.44	3.95	3.95	3.44	3.44	3.04	3.04	2.78	2.78

^1^Diets were formulated to be equivalent for SID lysine:NE for each of the dietary phases while differing in their NE.

^2^The vitamin and mineral premix provided the following per kilogram of diet: 8,000 IU vitamin A, 1,500 IU vitamin D, 30 IU vitamin E, 20 mg niacin, 12 mg D-pantothenic acid, 4 mg riboflavin, 2 mg menadione, 2 mg pyridoxine, 0.5 mg folic acid, 1 mg thiamin, 0.1 mg D-biotin, 0.02 mg vitamin B12, 100 mg Zn, 100 mg Fe, 15 mg Cu, 40 mg Mn; 1 mg I, 0.3 mg Se and 500 FYT Ronozyme HiPhos (DSM Nutritional Products Canada Inc., Ayr, ON, Canada).

^3^Digestible energy (DE) was calculated using the equations 1 to 3 from the NRC (2012): $\begin{array}{ll} & DE=4,168-(9.1\times ash)+(1.9\times crude\;protein)\backslash \end{array}$$\begin{array}{ll} & +\;(3.9\times ether\;extract)-(3.6\times neutral\;detergent\;f\;\;iber) \end{array}$.

^4^Net energy (NE) was calculated using the equations 1 to 8 from the NRC (2012): NE=(0.700×digestible energy)+(1.61×etherextract)+ (0.48×starch)−(0.91×crude protein)−(0.87×acid detergent fiber).

### Experimental Design

Three different dietary programs (low NE, medium NE, and high NE) were used in this study, each of which were fed to CON and IMP gilts. Thus, the experiment was designed as a 2 × 3 factorial with factors of Improvest management (CON or IMP) and dietary NE level (low NE, medium NE, or high NE). When expressed as a weighted average across the five dietary phases, the low NE treatment had an average NE level of 2,218 kcal/kg, the medium NE treatment had an average NE level of 2,343 kcal/kg, and the high NE treatment had an average NE level of 2,468 kcal/kg.

### Collection of On-Farm Data

The amount of feed delivered each day was measured using a robotic feeding system (FeedLogic). At the conclusion of each diet phase (days 26, 41, 54, 78, and 99), the amount of feed remaining was measured and used to calculate daily feed intake (*via* feed disappearance) for the given diet phase periods. Pen weights were collected at the start of the study, at the conclusion of each diet phase, and when gilts were marketed. Thus, weights were reported on day 0 (start of the study), day 26 (end of diet phase 1), day 41 (end of diet phase 2), day 54 (end of diet phase 3 and 1 d before the second Improvest dose), day 78 (end of diet phase 4 and 3 wk post-second Improvest dose), and when gilts were marketed (on days 83, 90, 97, and 104). Gain:Feed was calculated as average daily gain (ADG) divided by average daily feed intake (ADFI). Caloric intake and lysine intake were the product of formulated dietary composition and ADFI. Caloric intake:gain and lysine intake:gain were calculated as caloric intake divided by ADG and lysine intake divided by ADG, respectively.

It should be noted that marketing events began on day 83, thus growth performance calculations on day 83 and thereafter were reflective of the pigs remaining in the pen. Weights of the marketed pigs were collected on each of the marketing days. The number of pigs dead or removed from each pen throughout the study were recorded and growth performance calculations thereafter were reflective of the pigs remaining in the pen.

### Marketing and Slaughter Procedures

An equal number of gilts were marketed from each of the six treatment groups on day 83 (21.9% of the population), day 90 (21.9% of the population), day 97 (21.9% of the population), and day 104 (34.3% of the population) of the experiment. These days coincided with 28, 35, 42, and 49 days post-second Improvest dose. Thus, the weighted average for time post-second Improvest dose to marketing was 39.8 d.

Pigs were slaughtered under commercial conditions and identification was maintained throughout the slaughter process with a tattoo that reflected the pen number. HCW were recorded by processing plant personnel and carcass dressing percentage was calculated as HCW divided by weight at marketing. Grading probe measurements (i.e., backfat thickness and muscle depth) were collected online using the left side of carcasses by experienced operators from provincial grading authorities using a Destron PG-100 probe (International Destron Technologies). The grading probe was inserted perpendicularly at the grading site between the third and fourth last ribs, 7 cm off the split line according to Canadian grading standards (Pomar and Marcoux, 2003). Backfat thickness and muscle depth (i.e., loin depth) measurements were used to obtain the predicted lean yield value for each carcass using the following equation Bohrer et al., 2023


$$\begin{array}{l} \begin{array}{llllll} & Destron\;predicted\;lean\;yield\;(2023\;equation)\; & =89.16298(1.63023\times backfat\;thickness)\;\; & \;(0.42126\times muscle\;depth)+(0.01930\times backfat\;thickness^{2})\; & +(0.00308\times muscle\;depth^{2})\;\; & +\;(0.00369\times backfat\;thickness\times muscle\;depth) \end{array} \end{array}$$

where backfat thickness (mm) and muscle depth (mm) are collected at the grading site for each carcass.

### Statistical Analysis

There was a total of eight pen replicates for each treatment interaction (Improvest management × dietary treatment). Pen was considered as the experimental unit for all analyses. Live weights were analyzed with PROC MIXED of SAS (v. 9.4, SAS Inst. Inc.; Cary, NC, USA) as repeated measures over time with fixed effects of Improvest management, dietary treatment, and their interaction and a random effect of replication. An unstructured covariance structure was selected based on the analysis of the fit statistics. A slice statement was used to detect statistical differences at each time. All other parameters were analyzed with PROC MIXED of SAS with fixed effects of Improvest management, dietary treatment, and their interaction and a random effect of replication. Differences between Improvest management, dietary treatment, and their interaction were considered significant at *P* ≤ 0.05.

## Results

### Live Weights

#### Pre-Improvest period.

There were no significant interactions between Improvest management and NE (*P* ≥ 0.32) for live weight of gilts during the pre-Improvest period (second dose of Improvest was administered on day 55; Table 2). Additionally, there were no significant effects (*P* ≥ 0.63) for Improvest management during the pre-Improvest period. There were significant effects for NE during the pre-Improvest period. Gilts fed medium NE diets were 0.9 kg heavier (*P* = 0.02) compared with gilts fed high NE diets on day 26 of the study and gilts fed medium NE diets were 1.5 kg heavier (*P* = 0.03) compared with gilts fed high NE diets on day 54 of the study. Gilts fed low NE diets were intermediate in value and not significantly different from the other two NE treatments on both days 26 and 54 of the study.

**Table 2.** Effects of dietary net energy (NE) on live weights for market gilts managed with (IMP) or without Improvest (CON)^1^^,^^2^

	Improvest management			Net energy treatment				*P*-values		
	CON	IMP	SEM	Low NE	Medium NE	High NE	SEM	Improvest	Net energy	Interaction
Weights^3^^,^^4^										
Starting weight (day 0), kg	30.7	30.8	0.71	30.7	30.8	30.7	0.72	0.73	0.86	0.92
day 26, kg	58.1	58.2	0.94	58.1^xy^	58.6^x^	57.7^y^	0.94	0.83	0.02	0.32
day 41, kg	74.6	74.8	1.09	74.4	75.3	74.4	1.11	0.62	0.07	0.57
day 54, kg	88.6	88.8	1.17	88.5^xy^	89.6^x^	88.1^y^	1.20	0.63	0.03	0.56
day 78, kg	115.3^b^	119.4^a^	1.25	116.7	118.2	117.2	0.99	< 0.01	0.31	0.94
Live weight at marketing, kg	132.8^b^	137.8^a^	0.49	133.9^y^	136.8^x^	135.2^xy^	0.58	< 0.01	< 0.01	0.16
Days to marketing following last full pen weight^5^, days	19.0^a^	18.6^b^	0.91	18.7	18.8	18.9	0.91	0.03	0.74	0.13

^ab, xy^Least squares means within each row of main effects with different superscripts are significantly different (*P* < 0.05).

^1^Improvest is a gonadotropin-releasing factor (GnRF) analog-diphtheria toxoid conjugate product approved for temporary suppression of ovarian function and estrus in market gilts (Zoetis Canada Inc.); IMP gilts received the first dose of Improvest on day 20 of the study and the second dose of Improvest on day 55 of the study.

^2^Diets were formulated to be equivalent for SID lysine:NE for each of the dietary phases while differing in their NE; medium NE diets were a 50:50 blend of the low NE and high NE diets mixed at the farm; low NE treatment was an average NE level of 2,218 kcal/kg, medium NE treatment was an average NE level of 2,343 kcal/kg, high NE treatment was an average NE level of 2,468 kcal/kg.

^3^Gilts were approximately 11 wk old at the beginning of the study.

^4^An equal number of gilts were marketed from each of the six treatment groups on day 83 (21.9% of the population), day 90 (21.9% of the population), day 97 (21.9% of the population), and day 104 (34.3% of the population) of the study.

^5^The average number of days from the last full pen weight (day 78) to the day when all gilts were marketed.

#### Post-Improvest period.

There were no significant interactions between Improvest management and NE (*P* ≥ 0.16) for live weight of gilts during the post-Improvest period (Table 2). While not statistically significant, it should be noted that weight at marketing was numerically affected by the interaction between Improvest management and NE. The magnitude of change for live weight at marketing between IMP gilts and CON gilts was 3.4 kg when gilts were fed low NE diets (*P* = 0.03), 5.0 kg when gilts were fed medium NE diets (*P* < 0.01), and 6.4 kg when gilts were fed high NE diets (*P* < 0.01; least squares means for interactions are not shown).

There were significant effects on Improvest management during the post-Improvest period (Table 2). Improvest gilts were 4.1 kg heavier (*P* < 0.01) than CON gilts on day 78 of the study and were 5.0 kg heavier (*P* < 0.01) than CON gilts at marketing. Days to marketing (i.e., the average number of days from the last full pen weight [day 78 of the study] to the day when all pigs were marketed) was affected by Improvest management. On average, Improvest gilts were marketed 0.4 d sooner (*P* = 0.03) than CON gilts.

There were significant effects for NE during the post-Improvest period. Gilts fed low NE diets were 1.6 kg lighter (*P* < 0.01) compared with gilts fed medium NE diets at marketing (Table 2). Gilts fed high NE diets were intermediate in value and not significantly different from the other two NE treatments at marketing.

### Live Performance Calculations

Live performance parameters were reported for the pre-Improvest period (days 0 to 54), post-Improvest period (day 55 to marketing), and overall period (day 0 to marketing; Table 3); as well as for intervals between 0 to 3 wk post-second dose (day 55 to 78) and 3 wk post-second dose to marketing (day 79 to marketing; Table 4).

**Table 3.** Effects of dietary net energy (NE) on live performance (average daily feed intake, average daily gain, and G:F) for market gilts managed with (IMP) or without Improvest (CON)^1^^,^^2^

	Improvest management			Net energy treatment				*P*-values		
	CON	IMP	SEM	Low NE	Medium NE	High NE	SEM	Improvest	Net energy	Interaction
Pre-Improvest period (days 0 to 54)										
Average daily feed intake, g/d	2,457	2,441	427	2,526^x^	2,470^y^	2,350^z^	435	0.34	<0.01	0.09
Average daily gain, g/d	1,069	1070	86	1,064^xy^	1,085^x^	1,059^y^	95	0.84	0.03	0.27
Gain:Feed	0.466	0.470	0.004	0.449^y^	0.477^x^	0.478^x^	0.005	0.47	<0.01	0.66
Post-Improvest period (d 55—marketing)										
Average daily feed intake, g/d	3,362^b^	3,859^a^	418	3,684^x^	3,681^x^	3,467^y^	445	<0.01	<0.01	0.79
Average daily gain, g/d	1,091^b^	1,219^a^	75	1,132^y^	1,169^x^	1,163^xy^	91	<0.01	0.02	0.22
Gain:Feed	0.325^a^	0.316^b^	0.003	0.308^z^	0.318^y^	0.336^x^	0.003	<0.01	<0.01	0.51
Overall period (day 0—marketing)										
Average daily feed intake, g/d	2,833^b^	3,026^a^	361	3,003^x^	2,973^x^	2,813^y^	371	<0.01	<0.01	0.37
Average daily gain, g/d	1,078^b^	1,132^a^	59	1,092^y^	1,120^x^	1,102^xy^	67	<0.01	<0.01	0.20
Gain:Feed	0.381^a^	0.374^b^	0.003	0.364^z^	0.377^y^	0.392^x^	0.003	<0.01	<0.01	0.91

^ab, xyz^Least squares means within each row of main effects with different superscripts are significantly different (*P* < 0.05).

^1^Improvest is a gonadotropin-releasing factor (GnRF) analog-diphtheria toxoid conjugate product approved for temporary suppression of ovarian function and estrus in market gilts (Zoetis Canada Inc.); IMP gilts received the first dose of Improvest on day 20 of the study and the second dose of Improvest on day 55 of the study.

^2^Diets were formulated to be equivalent for SID lysine:NE for each of the dietary phases while differing in their NE; medium NE diets were a 50:50 blend of the low NE and high NE diets mixed at the farm; low NE treatment was an average NE level of 2,218 kcal/kg, medium NE treatment was an average NE level of 2,343 kcal/kg, high NE treatment was an average NE level of 2,468 kcal/kg.

**Table 4.** Effects of dietary net energy (NE) on live performance (average daily feed intake, average daily gain, and G:F) for market gilts managed with (IMP) or without Improvest (CON) during the post-second dose period^1^^,^^2^

	Improvest management			Net Energy Treatment				*P*-values		
	CON	IMP	SEM	Low NE	Medium NE	High NE	SEM	Improvest	Net energy	Interaction
0 to 3 wk post-second dose (days 55 to 78)										
Average daily feed intake, g/d	3,268^b^	3,655^a^	41	3,520^x^	3,535^x^	3,331^y^	43	<0.01	<0.01	0.68
Average daily gain, g/d	1,109^b^	1,279^a^	11	1,173	1,201	1,207	13	<0.01	0.19	0.64
Gain:Feed	0.340^b^	0.350^a^	0.004	0.333^y^	0.340^y^	0.362^x^	0.004	<0.01	<0.01	0.69
3 wk post-second dose to marketing (day 79 to marketing)										
Average daily feed intake, g/d	3,511^b^	4,196^a^	58	3,956^x^	3,923^x^	3,681^y^	62	<0.01	<0.01	0.97
Average daily gain, g/d	1,061^b^	1,121^a^	17	1,066	1,116	1,090	21	0.02	0.25	0.17
Gain:Feed	0.303^a^	0.268^b^	0.005	0.272^y^	0.287^xy^	0.298^x^	0.006	<0.01	<0.01	0.34

^ab, xy^Least squares means within each row of main effects with different superscripts are significantly different (*P* < 0.05).

^1^Improvest is a gonadotropin-releasing factor (GnRF) analog-diphtheria toxoid conjugate product approved for temporary suppression of ovarian function and estrus in market gilts (Zoetis Canada Inc.); IMP gilts received the first dose of Improvest on day 20 of the study and the second dose of Improvest on day 55 of the study.

^2^Diets were formulated to be equivalent for SID lysine:NE for each of the dietary phases while differing in their NE; medium NE diets were a 50:50 blend of the low NE and high NE diets mixed at the farm; low NE treatment was an average NE level of 2,218 kcal/kg, medium NE treatment was an average NE level of 2,343 kcal/kg, high NE treatment was an average NE level of 2,468 kcal/kg.

#### Pre-Improvest period.

There were no significant interactions between Improvest management and NE (*P* ≥ 0.09) for ADFI, ADG, or feed efficiency during the pre-Improvest period (Table 3). There were no significant main effects of Improvest management (*P* ≥ 0.26) for ADFI, ADG, or feed efficiency during the pre-Improvest period. There were significant effects of NE for ADFI, ADG, and feed efficiency during the pre-Improvest period. Gilts fed low NE and medium NE diets consumed more feed compared with the gilts fed high NE diets (7.5% greater ADFI for low NE and 5.1% greater ADFI for medium NE; *P* < 0.01) and pigs fed low NE diets consumed more feed compared to pigs fed medium NE diets (2.3% greater; *P* < 0.01) during the pre-Improvest period. Gilts fed medium NE diets grew faster compared with gilts fed high NE diets (2.4% greater ADG; *P* < 0.01), while gilts fed low NE diets were intermediate in value and not different compared with the other two NE treatments during the pre-Improvest period. This resulted in lower feed efficiency for gilts fed low NE diets compared with gilts fed medium NE and high NE diets (5.9% lower G:F compared with medium NE and 6.1% lower G:F compared with high NE; *P* < 0.01) during the pre-Improvest period.

#### Post-Improvest period.

There were no significant interactions between Improvest management and NE (*P* ≥ 0.22) for ADFI, ADG, or feed efficiency during the post-Improvest period (Table 3). There were significant effects for the main effects of both Improvest management and NE for ADFI, ADG, and feed efficiency during the post-Improvest period. Improvest gilts consumed more feed (14.8% greater ADFI; *P* < 0.01), grew faster (11.7% greater ADG; *P* < 0.01), and were less efficient (2.8% lower G:F; *P* < 0.01) compared with CON gilts during the post-Improvest period. Gilts fed low NE and medium NE diets consumed more feed compared with gilts fed high NE diets (6.3% greater ADFI for low NE and 6.2% greater ADFI for medium NE; *P* < 0.01) during the post-Improvest period. Gilts fed low NE diets grew slower compared with gilts fed medium NE diets (3.2% lower ADG; *P* < 0.01), while gilts fed high NE diets were intermediate in value and not different compared with the other two NE treatments during the post-Improvest period. This resulted in the lowest feed efficiency for gilts fed low NE diets (3.1% lower G:F compared with medium NE and 8.3% lower G:F compared with high NE; *P* < 0.01) during the post-Improvest period. Additionally, gilts fed medium NE diets were 5.4% less efficient compared with gilts fed high NE diets during the post-Improvest period (*P* < 0.01).

To further elucidate differences in ADFI, ADG, and feed efficiency, calculations for the first 3-wk interval post-second dose and for the 3-wk interval proceeding were evaluated (Table 4). There were no significant interactions between Improvest management and NE (*P* ≥ 0.17) for ADFI, ADG, or feed efficiency during either one of these intervals. There were significant effects of Improvest management for ADFI, ADG, and feed efficiency as well as significant effects of NE for ADFI and feed efficiency for each of the 3-wk intervals. Improvest gilts consumed more feed (11.8% greater ADFI; *P* < 0.01), grew faster (15.3% greater ADG; *P* < 0.01), and were more efficient (2.9% greater G:F; *P* < 0.01) compared with CON gilts during the first 3-wk interval of post-Improvest period (days 55 to 78). Improvest gilts consumed more feed (19.5% greater ADFI; *P* < 0.01), grew faster (5.7% greater ADG; *P* < 0.01), and were less efficient (11.6% lower G:F; *P* < 0.01) compared with CON gilts during the second 3-wk interval of post-Improvest period (day 79 to marketing). Gilts fed low NE and medium NE diets consumed more feed compared with gilts fed high NE diets during both 3-wk intervals (5.7% greater ADFI for low NE and 6.1% greater ADFI for medium NE during the first 3-wk interval and 7.5% greater ADFI for low NE and 6.6% greater ADFI for medium NE during the first 3-wk interval during the second 3-wk interval; *P* < 0.01). This resulted in gilts fed low NE and medium NE diets being less efficient compared with the gilts fed high NE diets during the first 3-wk interval (8.0% lower G:F for low NE and 6.1% lower G:F than medium NE; *P* < 0.01) and gilts fed low NE diets being less efficient compared with the gilts fed high NE diets during the second 3-wk interval (8.7% lower G:F for low NE; *P* < 0.01).

#### Overall period.

There were no significant interactions between Improvest management and NE (*P* ≥ 0.20) for ADFI, ADG, or feed efficiency during the overall period (day 0 to marketing; Table 3). There were significant effects of both Improvest management and NE for ADFI, ADG, and feed efficiency during the overall period. Improvest gilts consumed more feed (6.8% greater ADFI; *P* < 0.01), grew faster (5.0% greater ADG; *P* < 0.01), and were less efficient (1.8% lower G:F; *P* < 0.01) compared with CON gilts during the overall period. Gilts fed low NE and medium NE diets consumed more feed compared with gilts fed high NE diets (6.8% greater ADFI for low NE and 5.7% greater ADFI for medium NE; *P* < 0.01) during the overall period. Gilts fed low NE diets grew slower compared with gilts fed medium NE diets (2.5% lower ADG; *P* < 0.01), while gilts fed high NE diets were intermediate in value for ADG and not different compared with the other two NE treatments during the overall period. This resulted in poorer feed efficiency for gilts fed low NE diets compared with gilts fed medium NE and high NE diets (3.4% lower G:F compared with medium NE and 7.1% lower G:F compared with high NE; *P* < 0.01) and poorer feed efficiency for gilts fed medium NE diets compared with gilts fed high NE diets (3.8% lower G:F; *P* < 0.01)during the overall period.

### Caloric and Lysine Intake and Efficiency

Caloric intake, caloric intake:gain, lysine intake, and lysine intake:gain were reported for the pre-Improvest period (days 0 to 54), post-Improvest period (day 55 to marketing), and overall period (day 0 to marketing; Table 5); as well as for intervals between 0 to 3 wk post-second dose (days 55 to 78) and 3 wk post-second dose to marketing (day 79 to marketing; Table 6). Caloric intake:gain and lysine intake:gain were calculated as caloric intake divided by ADG and lysine intake divided by ADG, respectively. Therefore, greater values for caloric intake:gain and lysine intake:gain should be interpreted as a greater number of calories (kcal) or lysine (g SID) consumed to achieve a kg of ADG.

**Table 5.** Effects of dietary net energy (NE) on caloric and lysine intake and efficiency for market gilts managed with (IMP) or without Improvest (CON)^1^^,^^2^^,^^3^

	Improvest management			Net energy treatment				*P*-values		
	CON	IMP	SEM	Low NE	Medium NE	High NE	SEM	Improvest	Net energy	Interaction
Pre-Improvest period (days 0 to 54)										
Caloric intake, kcal NE/d	5,710	5,679	100	5,570^y^	5,749^x^	5,764^x^	102	0.39	< 0.01	0.17
Caloric intake:gain, kcal NE/kg gain	5,333	5,291	60	5,219^y^	5,289^y^	5,426^x^	62	0.25	< 0.01	0.86
Lysine intake, g SID lysine/d	22.91	22.78	0.40	22.38^y^	23.10^x^	23.06^x^	0.41	0.34	< 0.01	0.12
Lysine intake:gain, g SID lysine/kg gain	21.42	21.25	0.24	21.00^y^	21.28^y^	21.73^x^	0.25	0.25	< 0.01	0.85
Post-Improvest period (day 55—marketing)										
Caloric intake, kcal NE/d	7,986^b^	9,232^a^	108	8,340^y^	8,788^x^	8,700^x^	114	< 0.01	< 0.01	0.66
Caloric intake:gain, kcal NE/kg gain	7,377^b^	7,758^a^	105	7,479	7,609	7,614	110	<0.01	0.19	0.35
Lysine intake, g SID lysine/d	23.33^b^	26.93^a^	0.31	24.40^y^	25.65^x^	25.35^x^	0.33	<0.01	<0.01	0.67
Lysine intake:gain, g SID lysine/kg gain	21.53^b^	22.57^a^	0.30	21.83	22.18	22.14	0.31	< 0.01	0.26	0.36
Overall period (day 0—marketing)										
Caloric intake, kcal NE/d	6,745^b^	7,294^a^	99	6,830^y^	7,130^x^	7,098^x^	102	<0.01	<0.01	0.40
Caloric intake:gain, kcal NE/kg gain	6,262^b^	6,412^a^	76	6,247^y^	6,344^xy^	6,420^x^	78	<0.01	<0.01	0.60
Lysine intake, g SID lysine/d	23.10^b^	24.67^a^	0.35	23.29^y^	24.26^x^	24.10^x^	0.36	< 0.01	< 0.01	0.32
Lysine intake:gain, g SID lysine/kg gain	21.47^b^	21.85^a^	0.25	21.38^y^	21.69^xy^	21.92^x^	0.26	< 0.01	< 0.01	0.77

^ab, xy^Least squares means within each row of main effects with different superscripts are significantly different (*P* < 0.05).

^1^Caloric intake and lysine intake were the product of formulated dietary composition and average daily feed intake.

^2^Improvest is a gonadotropin-releasing factor (GnRF) analog-diphtheria toxoid conjugate product approved for temporary suppression of ovarian function and estrus in market gilts (Zoetis Canada Inc.); IMP gilts received the first dose of Improvest on day 20 of the study and the second dose of Improvest on day 55 of the study.

^3^Diets were formulated to be equivalent for SID lysine:NE for each of the dietary phases while differing in their NE; medium NE diets were a 50:50 blend of the low NE and high NE diets mixed at the farm; low NE treatment was an average NE level of 2,218 kcal/kg, medium NE treatment was an average NE level of 2,343 kcal/kg, high NE treatment was an average NE level of 2,468 kcal/kg.

**Table 6.** Effects of dietary net energy (NE) on caloric and lysine intake and efficiency for market gilts managed with (IMP) or without Improvest (CON) during the post-second dose period^1^^,^^2^^,^^3^

	Improvest management			Net energy treatment				*P*-values		
	CON	IMP	SEM	Low NE	Medium NE	High NE	SEM	Improvest	Net energy	Interaction
0 to 3 wk post-second dose (days 55 to 78)										
Caloric intake, kcal NE/d	7,689^b^	8,600^a^	96	7,849^y^	8,325^x^	8,261^x^	102	<0.01	<0.01	0.65
Caloric intake:gain, kcal NE/kg gain	6,940^a^	6,732^b^	78	6,707^y^	6,943^x^	6,858^xy^	85	<0.01	0.03	0.55
Lysine intake, g SID lysine/d	23.35^b^	26.11^a^	0.29	23.93^y^	25.27^x^	24.98^x^	0.31	<0.01	<0.01	0.66
Lysine intake:gain, g SID lysine/kg gain	21.07^a^	20.44^b^	0.24	20.45	21.08	20.74	0.26	<0.01	0.06	0.55
3 wk post-second dose to marketing (day 79 to marketing)										
Caloric intake, kcal NE/d	8,326^b^	9,954^a^	134	8,901^y^	9,317^x^	9,202^xy^	145	<0.01	0.01	0.72
Caloric intake:gain, kcal NE/kg gain	7,876^b^	8,929^a^	165	8,362	8,369	8,478	187	<0.01	0.83	0.29
Lysine intake, g SID lysine/d	23.31^b^	27.87^a^	0.38	24.92^y^	26.09^x^	25.77^xy^	0.41	<0.01	0.01	0.72
Lysine intake:gain, g SID lysine/kg gain	22.05^b^	25.00^a^	0.46	23.41	23.43	23.74	0.52	<0.01	0.83	0.29

^ab, xy^Least squares means within each row of main effects with different superscripts are significantly different (*P* < 0.05).

^1^Caloric intake and lysine intake were the product of formulated dietary composition and average daily feed intake.

^2^Improvest is a gonadotropin-releasing factor (GnRF) analog-diphtheria toxoid conjugate product approved for temporary suppression of ovarian function and estrus in market gilts (Zoetis Canada Inc.); IMP gilts received the first dose of Improvest on day 20 of the study and the second dose of Improvest on day 55 of the study.

^3^Diets were formulated to be equivalent for SID lysine:NE for each of the dietary phases while differing in their NE; medium NE diets were a 50:50 blend of the low NE and high NE diets mixed at the farm; low NE treatment was an average NE level of 2,218 kcal/kg, medium NE treatment was an average NE level of 2,343 kcal/kg, high NE treatment was an average NE level of 2,468 kcal/kg.

#### Pre-Improvest period.

There were no significant interactions between Improvest management and NE (*P* ≥ 0.12) for caloric intake, caloric intake:gain, lysine intake, or lysine intake:gain during the pre-Improvest period (Table 5). There were no significant differences (*P* ≥ 0.25) between IMP gilts and CON gilts for caloric intake, caloric intake:gain, lysine intake, or lysine intake:gain during the pre-Improvest period. There were significant differences for caloric intake, caloric intake:gain, lysine intake, and lysine intake:gain for the main effect of NE during the pre-Improvest period. Gilts fed low NE diets had lower caloric intake and lysine intake compared with gilts fed medium NE and high NE diets (3.1% lower caloric intake and 3.1% lower lysine intake compared with medium NE, and 3.4% lower caloric intake and 2.9% lower lysine intake compared with high NE; *P* < 0.01) during the pre-Improvest period. Gilts fed low NE and medium NE diets had lower levels of caloric intake:gain and lysine intake:gain compared with gilts fed high NE diets (3.8% lower caloric intake:gain and 3.4% lower lysine intake:gain for low NE, and 2.5% lower caloric intake:gain and 2.1% lower lysine intake:gain for Medium NE; *P* < 0.01) during the pre-Improvest period.

#### Post-Improvest period.

There were no significant interactions between Improvest management and NE (*P* ≥ 0.36) for caloric intake, caloric intake:gain, lysine intake, or lysine intake:gain during the post-Improvest period (Table 5). There were significant differences between IMP gilts and CON gilts for caloric intake, caloric intake:gain, lysine intake, and lysine intake:gain during the post-Improvest period. Improvest gilts had greater caloric intake (15.6% greater; *P* < 0.01), greater caloric intake:gain (5.2% greater; *P* < 0.01), greater lysine intake (15.4% greater; *P* < 0.01), and greater lysine intake:gain (4.8% greater; *P* < 0.01) compared with CON gilts during the post-Improvest period. Caloric intake and lysine intake were significantly affected by NE; however, caloric intake:gain and lysine intake:gain were unaffected (*P* ≥ 0.19) during the post-Improvest period. Gilts fed low NE diets had lower caloric intake and lysine intake compared with gilts fed medium NE and high NE diets (5.1% lower caloric intake and 4.9% lower lysine intake compared with medium NE, and 4.1% lower caloric intake and 3.7% lower lysine intake compared with high NE; *P* < 0.01) during the post-Improvest period.

There were no significant interactions between Improvest management and NE (*P* ≥ 0.29) for caloric intake, caloric intake:gain, lysine intake, or lysine intake:gain during the first 3-wk interval post-second dose (days 55 to 78) or for the 3-wk interval proceeding (day 79 to marketing; Table 6). During the first 3-wk interval post-second dose (days 55 to 78), IMP gilts had greater caloric intake (11.8% greater; *P* < 0.01), lower caloric intake:gain (3.0% lower; *P* < 0.01), greater lysine intake (11.8% greater; *P* < 0.01), and lower lysine intake:gain (3.0% lower; *P* < 0.01) compared with CON gilts. During the second 3-wk interval post-second dose (day 79 to marketing), IMP gilts had greater caloric intake (19.6% greater; *P* < 0.01), greater caloric intake:gain (13.4% greater; *P* < 0.01), greater lysine intake (19.6% greater; *P* < 0.01), and greater lysine intake:gain (13.4% greater; *P* < 0.01) compared with CON gilts. During the first 3-wk interval post-second dose (days 55 to 78), gilts fed low NE diets had lower caloric intake and lysine intake compared with gilts fed medium NE and high NE diets (5.7% lower caloric intake and 5.3% lower lysine intake compared with medium NE, and 5.0% lower caloric intake and 4.2% lower lysine intake compared with high NE; *P* < 0.01). Gilts fed low NE diets had lower caloric intake:gain compared with gilts fed medium NE diets during the first 3-wk interval post-second dose (3.4% lower caloric intake:gain; *P* = 0.03), while gilts fed High NE diets had intermediate values that were not different from the other two treatments. Lysine intake:gain was not significantly affected by NE during the first 3-wk interval post-second dose. During the second 3-wk interval post-second dose (day 79 to marketing), gilts fed low NE diets had lower caloric intake (4.5% lower; *P* < 0.01) and lower lysine intake (4.5% lower; *P* < 0.01) compared with gilts fed medium NE diets, while gilts fed high NE diets were intermediate and not different than the other treatments for these parameters. During the second 3-wk interval post-second dose, there were no significant differences for caloric intake:gain or lysine intake:gain attributed to NE.

#### Overall period.

There were no significant interactions between Improvest management and NE (*P* ≥ 0.32) for caloric intake, caloric intake:gain, lysine intake, or lysine intake:gain during the overall period (day 0 to marketing; Table 5). There were significant differences for the main effects of Improvest management and for NE for caloric intake, caloric intake:gain, lysine intake, and lysine intake:gain during the overall period. Improvest gilts had greater caloric intake (8.1% greater; *P* < 0.01), greater caloric intake:gain (2.4% greater; *P* < 0.01), greater lysine intake (6.8% greater; *P* < 0.01), and greater lysine intake:gain (1.8% greater; *P* < 0.01) compared with CON gilts during the overall period. Gilts fed low NE diets had lower caloric intake and lysine intake compared with gilts fed medium NE and high NE diets (4.2% lower caloric intake and 4.0% lower lysine intake compared with medium NE, and 3.8% lower caloric intake and 3.4% lower lysine intake compared with high NE; *P* < 0.01) during the overall period. Gilts fed low NE diets had lower caloric intake:gain and lysine intake:gain compared with gilts fed high NE diets (3.8% lower caloric intake:gain and 3.4% lower lysine intake:gain; *P* < 0.01), while gilts fed medium NE diets were intermediate and not different from the other treatment groups during the overall period.

### Carcass Traits

There were no significant interactions between Improvest management and NE (*P* ≥ 0.30) for HCW, carcass dressing percentage, backfat thickness, muscle depth, or predicted lean yield (Table 7). While not statistically significant (*P* = 0.30), it should be noted that HCW was numerically affected by the interaction between Improvest management and NE. The magnitude of change for HCW between IMP gilts and CON gilts was 2.6 kg when gilts were fed low NE (*P* = 0.05), 3.4 kg when gilts were fed medium NE (*P* < 0.01), and 4.5 kg when gilts were fed high NE (*P* < 0.01).

**Table 7.** Effects of dietary net energy (NE) on carcass traits for market gilts managed with (IMP) or without Improvest (CON)^1^^,^^2^

	Improvest management			Net energy treatment				*P*-values		
	CON	IMP	SEM	Low NE	Medium NE	High NE	SEM	Improvest	Net energy	Interaction
Hot carcass weight, kg	103.8^b^	107.3^a^	0.37	104.2^y^	106.3^x^	106.3^x^	0.44	<0.01	<0.01	0.30
Dressing percentage, %	78.19	77.90	0.17	77.83^y^	77.71^y^	78.61^x^	0.20	0.21	0.01	0.79
Backfat thickness, mm	18.2^b^	20.1^a^	0.40	19.1	19.0	19.3	0.45	<0.01	0.89	0.65
Muscle depth, mm	62.7	62.5	0.56	61.7	62.8	63.2	0.62	0.67	0.06	0.36
Destron predicted lean yield (2023 equation)^3^, %	55.87^a^	54.61^b^	0.26	55.23	55.29	55.20	0.30	<0.01	0.97	0.67

^ab, xy^Least squares means within each row of main effects with different superscripts are significantly different (*P* < 0.05).

^1^Improvest is a gonadotropin-releasing factor (GnRF) analog-diphtheria toxoid conjugate product approved for temporary suppression of ovarian function and estrus in market gilts (Zoetis Canada Inc.); IMP gilts received the first dose of Improvest on day 20 of the study and the second dose of Improvest on day 55 of the study.

^2^Diets were formulated to be equivalent for SID lysine:NE for each of the dietary phases while differing in their NE; medium NE diets were a 50:50 blend of the low NE and high NE diets mixed at the farm; low NE treatment was an average NE level of 2,218 kcal/kg, medium NE treatment was an average NE level of 2,343 kcal/kg, high NE treatment was an average NE level of 2,468 kcal/kg.

^3^The equation used for Destron predicted lean yield (2023 equation) was the following: $\begin{array}{llll} & =89.16298-(1.63023\times backfat\;thickness)-(0.42126\times muscle\;depth)\; & \;+\;(0.01930\times backfat\;thickness^{2})+(0.00308\times muscle\;depth^{2})\; & \;+\;(0.00369\times backfat\;thickness\times muscle\;depth) \end{array}$.

Overall, HCW was 3.5 kg greater (*P* < 0.01) for IMP gilts compared with CON gilts (Table 7). Carcass dressing percentage was not different (*P* = 0.21) between IMP gilts and CON gilts. Backfat thickness was 1.9 mm greater (*P* < 0.01) for IMP gilts compared to CON gilts and muscle depth was not different (*P* = 0.67) between IMP gilts and CON gilts. This resulted in lower predicted lean yield for IMP gilts compared with CON gilts (1.26 percentage unit difference; *P* < 0.01).

Hot carcass weight and carcass dressing percentage were significantly affected by NE while other carcass parameters were not significantly affected by NE (Table 7). Carcasses from gilts fed low NE diets were 2.1 kg lighter (*P* < 0.01) compared with carcasses from gilts fed medium NE and High NE diets, while HCW was not different between gilts fed medium NE and high NE diets. Carcasses from gilts fed low NE and medium NE diets had lower carcass dressing percentage compared with carcasses from gilts fed high NE diets (0.78% unit difference between low NE and high NE and 0.90 percentage unit difference between medium NE and high NE; *P* = 0.01).

## Discussion

The basis of the current study was to compare performance of market gilts managed with immunological suppression of ovarian function and estrus with conventional gilts when fed dietary programs with differing NE density, therefore the discussion herein will focus on the results for the interaction between Improvest management and NE.

### Live Performance

It was hypothesized that market gilts managed with Improvest would respond to the low NE diets in a different manner than conventional market gilts, as it has been shown that market gilts managed with Improvest have a drastic increase in feed intake compared with conventional gilts (Poulsen Nautrup et al., 2020; Vasquez-Hidalgo et al., 2023). The mechanistic action for Improvest in market gilts is thought to be similar to the mechanistic action for Improvest in male pigs. Downregulation of the appetite-suppressing activity of androgens and estrogens, which are produced by the ovaries during periods of estrus, occurs when the hypothalamic-pituitary-gonadal axis is blocked by the production of gonadotropin-releasing hormone (GnRH)-antibodies (Asarian and Geary, 2006; Van den Broeke et al., 2016; Van den Broeke et al., 2022). Typical Improvest response levels for ADFI were achieved in this study (IMP gilts had 14.8% greater ADFI and 11.7% greater ADG during the post-Improvest period); however, there were no significant interactions between Improvest management and NE for any of the traits or time points evaluated in this study. The lack of significant interactions between Improvest management and NE for most of the live performance traits in the current study including rate of gain and feed efficiency suggests that typical response levels for market gilts managed with Improvest vs. conventional gilts can be expected when fed dietary programs with NE ranging from 2,218 kcal/kg to 2,468 kcal/kg.

Feed intake of grow-finish pigs is generally assumed to be closely associated with dietary energy concentration (Henry, 1985; Patience et al., 2015; Velayudhan et al., 2015). When pigs are fed diets that are low in NE, greater levels of feed intake occur as a compensation to meet dietary energy requirements (Spurlock et al., 1997; Smith et al., 1999; Schinckel et al., 2012; Hong et al., 2016; Fang et al., 2019). It is generally assumed that pigs are capable of compensating in a manner in which similar caloric intake will be achieved regardless of dietary energy, that is until the digestive tract has reached a maximum capacity (Henry, 1985; Nyachoti et al., 2004; Quiniou and Noblet, 2012; Schinckel et al., 2012).

Interestingly, the current study did not fully support this assumption. Caloric intake differed across the NE treatments for both IMP gilts and CON gilts throughout the study. During the pre-Improvest period (days 0 to 54), gilts fed low NE diets had lower caloric intake compared with gilts fed medium NE and high NE diets (3.1% lower caloric intake compared with medium NE and 3.4% lower caloric intake compared with high NE; *P* < 0.01). During the post-Improvest period (day 55 to marketing), gilts fed low NE diets had lower caloric intake compared with gilts fed medium NE and high NE diets (5.1% lower caloric intake compared with medium NE and 4.1% lower caloric intake compared with high NE; *P* < 0.01; Interaction *P* = 0.66). It could be inferred, using the aforementioned assumption, that the digestive tracts of gilts had reached a maximum capacity very early in this study, thus inhibiting caloric intake for the gilts fed the low NE diets; however, this assumption should be further evaluated as caloric intake of IMP gilts far exceeded that of CON gilts at each NE level during the post-Improvest period (day 55 to marketing). Greater investigation is required to confirm this assumption when technologies capable of altering feed intake levels, such as suppression of ovarian function and estrus in female pigs, are implemented.

An additional matter that should be considered when formulating diets for market gilts managed with Improvest is the levels of amino acid intake (and particularly lysine intake) during the late finishing stages. For instance, IMP gilts had 13.4% greater lysine intake:gain compared with CON gilts during the second 3-wk interval post-second dose (day 79 to marketing). It is plausible to speculate that diets could be diluted during these production periods as a significant cost savings approach. However, careful consideration for timing of diet dilutions must be provided as IMP gilts had 3.0% lower lysine intake:gain compared with CON gilts during the first 3-wk interval post-second dose (days 55 to 78).

### Carcass Traits

The lack of significant interactions between Improvest management and NE for the carcass traits measured in the current study including HCW, carcass dressing percentage, backfat thickness, muscle depth, and predicted lean yield suggests that typical response levels for market gilts managed with Improvest vs. conventional gilts can be expected when fed dietary programs with NE ranging from 2,218 kcal/kg to 2,468 kcal/kg. While not statistically significant (*P* = 0.30), the magnitude of change for HCW between IMP gilts and CON gilts was 2.6 kg when gilts were fed low NE (*P* = 0.05), 3.4 kg when gilts were fed medium NE (*P* < 0.01), and 4.5 kg when gilts were fed high NE (*P* < 0.01). Thus, HCW should be closely monitored when managing market gilts with Improvest and feeding low NE diets, particularly if HCW is a primary driver of profitability.

## Conclusions

Overall, typical Improvest response levels for live performance and carcass characteristics were sustained at each of the NE treatments evaluated; however, consideration should be provided to the known production impacts of diets with differing levels of NE. In particular, the increase in feed consumption of Improvest females during the post-Improvest period could lead to the investigation of unique feeding strategies to best optimize profitability and performance.

## References

[CIT0001] AOAC. 2016. Official methods of analysis of AOAC international. 20th ed. Washington (DC): Association of Official Analytical Chemists, Inc.

[CIT0002] Asarian, L., and N. Geary. 2006. Modulation of appetite by gonadal steroid hormones. Philos. Trans. R. Soc. London, Ser. B 361:1251–1263. doi: 10.1098/rstb.2006.1860 16815802 PMC1642706

[CIT0003] Bohrer, B. M., W. L. Flowers, J. M. Kyle, S. S. Johnson, V. L. King, J. L. Spruill, D. P. Thompson, A. L. Schroeder, and D. D. Boler. 2014. Effect of gonadotropin releasing factor suppression with an immunological on growth performance, estrus activity, carcass characteristics, and meat quality of market gilts. J. Anim. Sci. 92:4719–4724. doi: 10.2527/jas.2014-7756 25149345

[CIT0004] Bohrer, B. M., Y. Wang, J. B. Dorleku, C. P. Campbell, and I. B. Mandell. 2023. Technical note: an update of the predicted lean yield equation for the Destron PG-100 optical grading probe. J. Anim. Sci. 101:skad199. doi: 10.1093/jas/skad199 37317891 PMC10313092

[CIT0005] Canadian Council on Animal Care. 2009. CCAC guidelines on: the care and use of farm animals in research, teaching, and testing. Ottawa (ON): Canadian Council on Animal Care. https://ccac.ca/Documents/Standards/Guidelines/Farm_Animals.pdf

[CIT0006] Fang, L. H., Y. H. Jin, S. H. Do, J. S. Hong, B. O. Kim, T. H. Han, and Y. Y. Kim. 2019. Effects of dietary energy and crude protein levels on growth performance, blood profiles, and carcass traits in growing-finishing pigs. J. Anim. Sci. Technol. 61:204–215. doi: 10.5187/jast.2019.61.4.204 31452907 PMC6686147

[CIT0007] Henry, Y. 1985. Dietary factors involved in feed intake regulation in growing pigs: a review. Livest. Prod. Sci. 12:339–354. doi: 10.1016/0301-6226(85)90133-2

[CIT0008] Hong, J. S., G. I. Lee, X. H. Jin, and Y. Y. Kim. 2016. Effect of dietary energy levels and phase feeding by protein levels on growth performance, blood profiles and carcass characteristics in growing-finishing pigs. J. Anim. Sci. Technol. 58:37. doi: 10.1186/s40781-016-0119-z 27795835 PMC5075758

[CIT0009] Just, A. 1982. The net energy value of balanced diets for growing pigs. Livest. Prod. Sci. 8:541–555. doi: 10.1016/0301-6226(82)90032-x

[CIT0011] Noblet, J., S. Wu, and M. Choct. 2022. Methodologies for energy evaluation of pig and poultry feeds: a review. Anim. Nutr. 8:185–203. doi: 10.1016/j.aninu.2021.06.015 34977388 PMC8685914

[CIT0012] NRC. 2012. Nutritional requirements of swine. 11th rev. ed. Washington (DC): National Academic Press.

[CIT0013] Nyachoti, C. M., R. T. Zijlstra, C. F. M. De Lange, and J. F. Patience. 2004. Voluntary feed intake in growing-finishing pigs: a review of the main determining factors and potential approaches for accurate predictions. Can. J. Anim. Sci. 84:549–566. doi: 10.4141/a04-001

[CIT0014] Patience, J. F., M. C. Rossoni-Serão, and N. A. Gutiérrez. 2015. A review offeed efficiency in swine: biology and application. J. Anim. Sci. Biotechnol. 6:1–9. doi: 10.1186/s40104-015-0031-2 PMC4527244 26251721

[CIT0015] PIC. 2021. PIC® nutrition and feeding guidelines. Hendersonville, TN: PIC Genetics. https://www.pic.com/resources

[CIT0016] Pomar, C., and M. Marcoux. 2003. Comparing the Canadian pork lean yields and grading indexes predicted from grading methods based on Destron and Hennessy probe measurements. Can. J. Anim. Sci. 83:451–458. doi: 10.4141/A02-107

[CIT0017] Poulsen Nautrup, B., I. Van Vlaenderen, and C. K. Mah. 2020. The effect of immunization against gonadotropin-releasing factor in market gilts: meta-analyses of parameters relevant for pig producers, pork packers and retailers/consumers. Res. Vet. Sci. 131:159–172. doi: 10.1016/j.rvsc.2020.04.012 32387811

[CIT0018] Quiniou, N., and J. Noblet. 2012. Effect of the dietary net energy concentration on feed intake and performance of growing-finishing pigs housed individually. J. Anim. Sci. 90:4362–4372. doi: 10.2527/jas.2011-4004 22696619

[CIT0019] Schinckel, A. P., M. E. Einstein, S. Jungst, J. O. Matthews, C. Booher, T. Dreadin, C. Fralick, E. Wilson, and R. D. Boyd. 2012. Daily feed intake, energy intake, growth rate and measures of dietary energy efficiency of pigs from four sire lines fed diets with high or low metabolizable and net energy concentrations. Asian-Australas. J. Anim. Sci. 25:410–420. doi: 10.5713/ajas.2011.11212 25049580 PMC4092956

[CIT0020] Smith, J. W., M. D. Tokach, P. R. O’Quinn, J. L. Nelssen, and R. D. Goodband. 1999. Effects of dietary energy density and lysine: calorie ratio on growth performance and carcass characteristics of growing-finishing pigs. J. Anim. Sci. 77:3007–3015. doi: 10.2527/1999.77113007x 10568471

[CIT0021] Spurlock, M. E., G. R. Frank, G. M. Willis, J. L. Kuske, and S. G. Cornelius. 1997. Effect of dietary energy source and immunological challenge on growth performance and immunological variables in growing pigs. J. Anim. Sci. 75:720–726. doi: 10.2527/1997.753720x 9078489

[CIT0028] Van den Broeke, A., Aluwé, M., Kress, K., Stefanski, V., Škrlep, M., Batorek, N., Ampe, B., and Millet, S. 2022. Effect of dietary energy level in finishing phase on performance, carcass and meat quality in immunocastrates and barrows in comparison with gilts and entire male pigs. *Animal*, 16(1):100437. doi: 10.1016/j.animal.2021.100437 35007882

[CIT0027] Van den Broeke, A., Leen, F., Aluwé, M., Ampe, B., Van Meensel, J., and Millet, S. 2016. The effect of GnRH vaccination on performance, carcass, and meat quality and hormonal regulation in boars, barrows, and gilts. *J. Anim. Sci.* 94(7):2811–2820. doi: 10.2527/jas.2015-0173 27482668

[CIT0022] Vasquez-Hidalgo, M. A., M. A. Mellencamp, D. Amodie, L. G. Pantoja, and K. A. Vonnahme. 2023. The effect of timing of Improvest administration on growth performance and carcass characteristics in gilts. Transl. Anim. Sci. 7:txad051. doi: 10.1093/tas/txad051 37786423 PMC10541850

[CIT0023] Vasquez-Hidalgo, M. A., K. A. Vonnahme, S. Pollmann, D. Amodie, L. G. Pantoja, and M. Mellencamp. 2022. Improvest improves gilt performance. J. Anim. Sci. 100:30–31. doi: 10.1093/jas/skac064.049

[CIT0024] Velayudhan, D. E., I. H. Kim, and C. M. Nyachoti. 2015. Invited review - Characterization of dietary energy in swine feed and feed ingredients: a review of recent research results. Asian-Australas. J. Anim. Sci. 28:1–13. doi: 10.5713/ajas.14.0001R 25557670 PMC4283177

[CIT0025] Woyengo, T. A., E. Beltranena, and R. T. Zijlstra. 2014. Nonruminant nutrition symposium: controlling feed cost by including alternative ingredients into pig diets: a review. J. Anim. Sci. 92:1293–1305. doi: 10.2527/jas.2013-7169 24492540

[CIT0026] Zuidhof, M. 2019. A review of dietary metabolizable and net energy: uncoupling heat production and retained energy. J. Appl. Poult. Res. 28:231–241. doi: 10.3382/japr/pfx062

